# Occurrence and molecular characterization of *Giardia duodenalis* and *Cryptosporidium* spp. in sheep and goats reared under dairy husbandry systems in Greece☆

**DOI:** 10.1051/parasite/2014048

**Published:** 2014-09-05

**Authors:** Nikolaos Tzanidakis, Smaragda Sotiraki, Edwin Claerebout, Amimul Ehsan, Nikolaos Voutzourakis, Despoina Kostopoulou, Casaert Stijn, Jozef Vercruysse, Thomas Geurden

**Affiliations:** 1 Veterinary Research Institute – Hellenic Agricultural Organization Demeter 57001 Thermi, Thessaloniki Greece; 2 Laboratory for Parasitology, Faculty of Veterinary Medicine, Ghent University Salisburylaan 133 9820 Merelbeke Belgium

**Keywords:** *Giardia*, *Cryptosporidium*, Goat, Sheep, Dairy, Prevalence, Genotyping

## Abstract

*Giardia duodenalis* and *Cryptosporidium* spp. are gastro-intestinal protozoa known to infect small ruminants. Both protozoa are also considered as a potential public health concern. The objective of this study was to determine their prevalence in lambs and goat kids kept under common Mediterranean dairy husbandry systems and to identify the species and genotypes infecting these small ruminants. In total, 684 faecal samples (429 from lambs and 255 from goat kids) were collected on 21 farms in Greece and examined using a quantitative immunofluorescence assay. *G. duodenalis* was detected in 37.3% of the lambs and 40.4% of the goat kids. On all but one of the farms *G. duodenalis* was detected. Most samples were typed as a mono-infection with *G. duodenalis* assemblage E, both on the β-giardin gene and the triose phosphate isomerase gene. Only 10% of samples were typed as mixed assemblage A and E infections. The prevalence of *Cryptosporidium* spp. was 5.1% in lambs and 7.1% in goat kids. In total, 8 out of the 14 farms with a sheep flock and 7 out of the 14 farms with a goat flock were positive. *Cryptosporidium parvum* (subtype IId), *C. ubiquitum* and *C. xiaoi* were identified, the latter especially in goat kids. In conclusion, the results of the present study illustrate that *G. duodenalis* and *Cryptosporidium* spp. occur frequently on both sheep and goats farms. The prevalence of zoonotic genotypes or species was low, indicating a limited but existing risk for zoonotic infections.

## Abstract (fr)

*Giardia duodenalis* et *Cryptosporidium* spp. sont des protozoaires gastro-intestinaux connus pour infecter les petits ruminants. Les deux protozoaires sont également considérés comme un risque potentiel pour la santé publique. L’objectif de cette étude était de déterminer la prévalence chez les agneaux et les chevreaux élevés dans les systèmes méditerranéens communs d’élevage laitier et d’identifier les espèces et les génotypes infectant ces petits ruminants. Au total, 684 échantillons fécaux (429 provenant d’agneaux et 255 de chevreaux) ont été collectés dans 21 exploitations en Grèce et examinés à l’aide d’un test quantitatif d’immunofluorescence. *G. duodenalis* a été détecté chez 37.3 % des agneaux et 40.4 % des chevreaux, dans toutes les fermes sauf une. La plupart des échantillons ont été typés comme une mono infection par l’assemblage E de *G. duodenalis*, en se fondant à la fois sur l’identification du gène de la β-giardine ou du gène de l’isomérase de triose phosphate. Seuls 10 % des échantillons ont été identifiés comme une combinaison d’infections par les assemblages A et E. La prévalence de *Cryptosporidium* spp. était de 5.1 % chez les agneaux et de 7.1 % chez les chevreaux. Au total, 8 des 14 fermes ovines et 7 des 14 fermes caprines étaient positives. *Cryptosporidium parvum* (sous-type IId), *C. ubiquitum* et *C. xiaoi* ont été identifiés, ce dernier en particulier chez les chevreaux. En conclusion, les résultats de cette étude montrent que *G. duodenalis* et *Cryptosporidium* spp. sont fréquemment présents chez les ovins et les caprins. La prévalence des génotypes ou espèces zoonotiques était faible, ce qui indique un risque limité bien que présent pour des infections zoonotiques.

## Introduction

*Giardia duodenalis* and *Cryptosporidium* spp. are gastro-intestinal protozoa that affect a wide range of mammals. Both parasites have a direct life cycle and are known to cause enteritis. In small ruminants, mainly young lambs and goat kids are infected. The prevalence of both parasites in small ruminants varies considerably between studies worldwide [13]. The most common clinical symptoms associated with *G. duodenalis* are the excretion of malodorous, loose to diarrhoeic faeces and impaired weight gain, whereas *Cryptosporidium* spp. infection can lead to severe diarrhoea, depression, anorexia and weight loss [1, 12]. Mortality has been associated with cryptosporidiosis, especially in animals with concurrent infections.

Since the initial claims on its potential public health relevance, eight genotypes or so-called assemblages have been identified within *G. duodenalis* [25]. Assemblage A and B are considered to be zoonotic genotypes, affecting both humans and small ruminants [1, 12, 16, 23], whereas Assemblage E is considered to be specific to hoofed livestock and has been found to be the most prevalent assemblage in lambs and goat kids [8, 12]. The genus *Cryptosporidium* consists of 20 species and more than 40 genotypes. In sheep, *C. parvum*, *C. xiaoi* and *C. ubiquitum* are most frequently identified [9, 12, 27, 29, 35, 41] whereas mainly *C. parvum* and to a lesser extent *C. xiaoi* has been reported in goats [9, 12, 35]. Due to the potential clinical and zoonotic relevance, there is a need to better understand the presence and abundance of both parasites in sheep and goat flocks.

The aim of this study was to estimate the prevalence of *G. duodenalis* and *Cryptosporidium* spp. infection in sheep and goat dairy farms in Greece. The small ruminant sector is very important in the Mediterranean basin from an economic, social and ecological point of view [5], especially in Greece with approximately 15 million sheep and goats which are kept traditionally for milk under low-input systems [20]. The most commonly applied farming systems practised in Greece can be categorized as extensive farming [43], and there is limited information on prevalence and especially molecular characterization of both parasites in these systems. The prevalence rates reported range from 33.3% to 49.6% for *G. duodenalis* and from 4.4% to 55% for *Cryptosporidium* spp. in sheep and goats [21, 32].

## Materials and methods

### Study design

The study was designed as a cross-sectional study in a high sheep and goat density area in Greece (i.e. the island of Crete where more than 1.5 million animals are kept). The farms enrolled in this survey were selected according to the following criteria: (a) type of animal on the farm (sheep, goats, or mixed sheep and goats), and (b) management practices applied (“intensive management system” where stocking rates are high and the young animals are reared indoors until weaning (30–40 days), and “extensive management system” where stocking rates are lower and young animals are reared with their mothers mostly on pasture). Each farm was visited on a single occasion in a 6-month period, and only animals between the age of 1 day and 10 weeks were considered for inclusion in the study. Sample size was calculated based on the number of expected births as an indicator of the number of animals on the farms: of the lambs, 5% of the expected births on each farm were sampled and of the goat kids, 10% of the expected births were sampled. Data on the type of water supply for the animals (public network supply, a private drill hole or natural well), the age of lambs or goat kids and the presence of diarrhoea were recorded.

### Parasitological examination

The faecal samples were examined in the laboratory using a quantitative immunofluorescence assay (IFA; Merifluor *Cryptosporidium/Giardia* kit; Meridian Diagnostics Inc.), as follows: 1 g of faeces was suspended in tap water and sieved three times through a layer of surgical gauze to withhold large debris. Sedimentation for at least 30 min was followed by discarding the supernatant. The remaining sediment was centrifuged at 3000 rpm for 5 min. The sediment was re-suspended in 1 mL of tap water. After thorough vortexing, an aliquot of 20 μL was applied onto an IFA slide. The samples, including a negative and positive control sample, were left to dry completely. After staining and incubating slides in a dark humidified chamber (for 30 min at room temperature), the entire slide was examined at 400× magnification under a fluorescence microscope. A sample was considered positive if at least one, clearly recognizable *Cryptosporidium* oocyst or *Giardia* cyst was identified. The number of (oo)cysts per gram of faeces was obtained by multiplying the total number of (oo)cysts on the slide by 50.

### Molecular characterization

Positive isolates for both parasites were selected for DNA extraction, using the QIAamp Stool Mini Kit (Qiagen), according to the manufacturer’s instructions, incorporating an extended initial step of five freeze-thaw cycles (freezing in liquid nitrogen for 5 min and heating at 95 °C for 5 min) in the protocol to maximize (oo)cyst lesion. The selection of the positive isolates aimed to include at least one positive sample per farm.

For the amplification of the *Cryptosporidium* spp., the 18S ribosomal DNA (18S rDNA) gene PCR protocol was used (previously described in [40]), as well as a PCR targeting the 70 kDA heat shock protein (HSP70, described in [28]). Subgenotyping of the *C. parvum* positive samples was performed using the 60 kDa glycoprotein (gp60) gene [34]. *G. duodenalis* positive samples were characterized using the β-giardin gene [24], and the triose phosphate isomerase (TPI) gene [11]. Amplification products were visualized on 1.5% agarose gels with ethidium bromide. A positive and negative (PCR water) control sample was included in each PCR. PCR products were purified using the Qiaquick purification kit (Qiagen) and fully sequenced using the BigDye Terminator V3.1 Cycle Sequencing Kit (Applied Biosystems). Sequencing reactions were analysed on a 3100 Genetic Analyzer (Applied Biosystems) and assembled with Seqman II (DNASTAR, Madison, WI, USA). Sequences were compared with known sequences by BLAST-analysis against the NCBI database.

## Results

A total of 21 farms with either a sheep (*n* = 7) or goat (*n* = 7) flock, or a mixed sheep and goat flock (*n* = 7) were visited. On the majority of the farms (14/21) animals were reared under the intensive management system, whereas on 7 farms, goats and sheep were reared under the extensive management system (Table 1). In total, 684 faecal samples were examined, of which 429 samples were from lambs and 255 from goat kids. Mean lamb age was 5 weeks (range 4–8 weeks) and mean goat kid age was 6 weeks (range 4–9 weeks).

**Table 1.** Overview of the 21 farms included in the study, with the number of sheep and/or goats on the farms (N) and the type of management system (Sampled = number of animals sampled on each farm).

Farm identification number	Management system	Sheep		Goats	
		Number of animals	Sampled	Number of animals	Sampled
1	Intensive	203	17	0	0
7	Intensive	500	38	0	0
10	Intensive	450	29	0	0
11	Extensive	550	41	0	0
14	Extensive	170	13	0	0
16	Extensive	100	13	0	0
18	Extensive	750	34	0	0
2	Intensive	656	49	40	6
19	Extensive/intensive	400	24	40	8
4	Intensive	301	23	68	11
9	Intensive	500	38	85	14
3	Intensive	500	16	100	16
8	Intensive	680	51	174	28
12	Extensive	570	43	240	38
24	Extensive	0	0	190	15
22	Intensive	0	0	200	16
25	Intensive	0	0	210	17
27	Intensive	0	0	215	17
23	Intensive	0	0	250	20
26	Intensive	0	0	300	24
21	Intensive	0	0	315	25
	Total		429		255

The prevalence of *G. duodenalis* was 37.3% (*n* = 160/429) in lambs and 40.4% (*n* = 103/255) in goat kids. In total, 13 out of the 14 farms (92.9%) with a sheep flock, and 14 out of the 14 farms (100%) with a goat flock, were positive for *G. duodenalis* with a minimum of 4 positive samples on each positive farm. The prevalence of *Cryptosporidium* spp. oocysts was 5.1% (*n* = 22/429) in lambs and 7.1% (*n* = 18/255) in goat kids. In total, 8 out of the 14 farms (57.1%) with a sheep flock, and 7 out of the 14 farms (50.0%) with a goat flock, were positive with a minimum of 1 positive sample on each positive farm.

Intensity of *G. duodenalis* cyst excretion ranged from 50 to 800,000 cysts per gram (cpg) of faeces in lambs with an average of 48,989 cpg, and from 50 to 900,000 cpg for goat kids with an average of 94,053 cpg. The excretion level for *Cryptosporidium* oocysts was low in lambs with an average of 9143 oocysts per gram of faeces (range 200–31,900 opg), yet was high in goat kids with an average excretion of 47,744 opg (range 200–551,000 opg).

A minimum of 1 positive sample was selected for DNA extraction per farm for *G. duodenalis* and *Cryptosporidium* spp., respectively. In total, 71 samples yielded a positive amplicon for *G. duodenalis* (Table 2). The majority of the samples were typed as a mono-infection with the ruminant-specific assemblage E, both on the β-giardin gene and the TPI gene. Only a limited number of samples were typed as mixed assemblage A and E infections, both in lambs and in goat kids. For *Cryptosporidium*, 24 samples yielded a positive amplicon (Table 3). Three different *Cryptosporidium* species (*C. parvum*, *C. ubiquitum* and *C. xiaoi*) were identified, although *C. xiaoi* was not identified in lambs. The *C. parvum* positive samples were typed as subtype IId on the gp60 gene (IIdA4G2T14 and IIdA4G3T13).

**Table 2.** Results for molecular identification of *Giardia duodenalis* positive samples in goat kids and lambs, based on the B-giardin (= beta-giardin) or triose phosphate isomerase (TPI) gene (NA = no amplification; A = assemblage A; E = assemblage E; A + E = mixed infection with assemblage A and E).

Animal species	Number of samples	B-giardin			TPI			
		A	E	NA	A	E	A + E	NA
Goat kids	30	1	28	1	1	26	3	0
Lambs	41	1	35	5	1	35	3	2
Total	71	2	63	6	2	61	6	2

**Table 3.** Results for molecular identification of *Cryptosporidium* positive samples in goat kids and lambs.

Animal species	Number of samples	*C. xiaoi*	*C. ubiquitum*	*C. parvum*
Goat kids	14	7	5	2 (IId)
Lambs	10	0	3	7 (IId)
Total	24	7	8	9

## Discussion

The present study illustrates that *G. duodenalis* is highly prevalent in both lambs and goat kids, as the parasite was detected in all but one of the sheep flocks and in all goat flocks. The high farm and animal prevalence is in line with previous studies in small ruminants in Europe [4, 13, 18]. The high *G. duodenalis* prevalence and the potential association with production losses [31, 44] require an appropriate level of awareness of this infection on those farms in terms of disease management and prevention of infection. In contrast to *G. duodenalis*, the prevalence of *Cryptosporidium* was lower than anticipated in both lambs and goat kids, probably due to the age range of the animals included in the present study. Nevertheless, the farm prevalence on the sheep (57.1%) and goat (50.0%) farms does suggest that *Cryptosporidium* is widespread and is a potential threat to the small ruminant population. In previous studies in Greece, *Cryptosporidium* has been associated with large outbreaks of diarrhoea in both sheep and goat flocks [14, 15, 37], similar to other major small ruminant rearing countries [6, 33].

Both for *G. duodenalis* and for *Cryptosporidium* spp. a potential public health threat has been suspected, based on the high prevalence of these parasites in small ruminants and on extrapolation of molecular insights from other animal species, such as cattle or companion animals. However, recent molecular data seem to suggest that small ruminants are mostly infected with non-zoonotic genotypes of *G. duodenalis* [12, 18, 38, 39, 46, 47] and *Cryptosporidium* spp. [38]. On the other hand, potentially zoonotic genotypes or species such as *G. duodenalis* assemblage A and B [1, 2, 12, 19, 26, 30, 41], *C. parvum* [3, 6, 12, 22, 46], *C. hominis* [17], *C. meleagridis* [42] and *C. ubiquitum* [10, 12, 45] have been reported in small ruminants. Furthermore, the identification of potentially zoonotic genotypes does not necessarily imply that transmission occurs. Recently, host-associated populations of *C. parvum* have been described using a multi-locus genotyping (MLG) approach, and *C. parvum* populations found in goats were even found to differ from bovine and sheep MLGs [7]. Whether this is a true host-specific phenomenon or just a matter of the level of isolation and opportunities for out-crossing is still to be discussed. Nevertheless, the contradicting molecular findings illustrate the difficulty of evaluating a potential public health threat based solely on genetic data without considering the epidemiological background and transmission of infection. Direct transmission of *Cryptosporidium* infection through bottle feeding or petting of animals on educational farms has been described before [38], but is probably an occasional route of infection. Although there is no direct evidence of transmission of *Cryptosporidium* and *G. duodenalis* infections from small ruminants to the human population via contaminated water, it is considered a threat. Furthermore, the detection of both parasites in outbreaks and in water screening is not routine practice in most countries, and large waterborne outbreaks might go unnoticed. In a recent study in Spain, the prevalence of *Cryptosporidium* and *Giardia* in water was significantly higher in the inland area, with higher concentration of livestock and fewer water treatment plants [4], illustrating that a variety of factors define the odds for infection. In the specific study area on the island of Crete, only a limited number of water basins are used over the island to produce drinking water for the local population and for the tourist population in the summer. The pastures surrounding the drinking water basins are all grazed by small ruminants. Whether these conditions lead to a substantial public health threat will need to be evaluated further in a longitudinal study, including sampling of water.

In goats, a large proportion of the *Cryptosporidium* positive samples were typed as *C. xiaoi*, both on 18S and HSP-70. This is in agreement with previous studies in Spain [6] and France [36], yet contradicts the initial claim that *C. xiaoi* infections are largely restricted to sheep [9]. In the current study, *C. xiaoi* was found in goats from three different farms, of which 2 farms maintained a goat-only flock and 1 farm managed a mixed flock. This illustrates that, although the introduction in the goat flocks might be due to contact with infected sheep, a *C. xiaoi* infection is easily maintained in goats. As advocated by Fayer and Santin [9], further epidemiological data will be needed to confirm whether the reports of *C. xiaoi* in goats are incidental or a regularly observed finding.

In conclusion, a high animal and farm prevalence of *G. duodenalis*, and a high farm prevalence of *Cryptosporidium* spp. were detected in both lambs and goat kids. Although mainly non-zoonotic species were identified in the present study, the frequent contact and proximity of grazing grounds to the natural water sources used to produce drinking water in the study area warrant further investigation of the public health relevance of these infections.
